# Affective prosody and cortical activation in dementia of the Alzheimer’s type: an exploratory acoustic and fNIRS study

**DOI:** 10.3389/frdem.2025.1681602

**Published:** 2025-10-09

**Authors:** Chorong Oh, In-Sop Kim, Ann Feltis

**Affiliations:** ^1^Department of Communication Sciences and Disorders, University of Kentucky, Lexington, KY, United States; ^2^Department of Hearing, Speech & Language Sciences, Ohio University, Athens, OH, United States; ^3^School of Allied Health & Communicative Disorders, Northern Illinois University, DeKalb, IL, United States

**Keywords:** dementia, speech, acoustics, fNIRS, emotion

## Abstract

Affective prosody, the expression of emotion via speech, is critical for successful communication. In dementia of the Alzheimer’s type (DAT), impairments in expressive prosody may contribute to interpersonal difficulties, yet the underlying acoustic and neural mechanisms are not well understood. This exploratory study examined affective prosody production and cortical activation in individuals with DAT using a multimodal approach. Ten participants with DAT completed three speech tasks designed to elicit happy, sad, and neutral emotional tones. Acoustic features were extracted using Praat software, and cerebral hemodynamics were recorded using functional near-infrared spectroscopy (fNIRS), focusing on oxygenated (HbO) and total (HbT) hemoglobin levels across hemispheres. Multinomial logistic regression showed that initial fundamental frequency and speech rate significantly predicted emotional condition. Paired-sample *t*-tests revealed hemispheric differences in HbO and HbT during affective speech, particularly in the happy and neutral conditions. These findings suggest that individuals with DAT may exhibit reduced modulation of affective prosody and altered patterns of hemispheric activation during emotionally expressive speech. While preliminary and limited by the absence of a control group, this study highlights behavioral and neural features that may contribute to communication challenges in DAT and provides a foundation for future research on affective prosody as a potential target for intervention or monitoring.

## 1 Introduction

Affective prosody, the modulation of pitch, loudness, tempo, and rhythm to express emotion, is fundamental to effective communication. It conveys emotional intent beyond the literal meaning of speech and plays a key role in social interaction. Prosody is commonly divided into two types: linguistic and affective. While linguistic prosody supports syntactic and pragmatic cues, affective prosody expresses the speaker’s emotional state (Banse and Scherer, 1996; Beach, 1991).

Prosodic impairments have been observed across a range of neurological and communication disorders, including aphasia, Parkinson’s disease, and traumatic brain injury. In aphasia, disruptions in both expressive and receptive prosody have been documented, reflecting damage to right-hemisphere or interhemispheric networks (Baum and Pell, 1999; Patel et al., 2018; Ross and Monnot, 2008). Similarly, dysprosody in Parkinson’s disease has been consistently reported and linked to motor and basal ganglia dysfunction (Harel et al., 2004; Skodda et al., 2010; Pell et al., 2006). Findings from traumatic brain injury and other neuropathologies further underscore prosody’s vulnerability to diffuse neural damage (Ilie et al., 2017; Bornhofen and McDonald, 2008; Belyk and Brown, 2017). Together, these literatures highlight prosody as a sensitive marker of neuropathology more broadly.

Within this broader context, dementia of the Alzheimer’s type (DAT), the most common form of dementia, represents a particularly important case, as prosodic changes may provide insight into both communication impairments and underlying neural dysfunction. Although primarily associated with cognitive decline, dementia has increasing been linked to prosodic impairments. Affective prosody, in particular, engages distributed interhemispheric networks that are often compromised in DAT (Lian et al., 2020; Qi et al., 2019). However, how individuals with DAT express affective prosody and the associated neural mechanisms remain poorly understood. Most prior studies on prosodic changes in individuals with dementia have focused on the perception of affective prosody, yielding mixed results. Some report impaired comprehension of affective prosody in DAT and frontotemporal dementia (Taler et al., 2008; Rohrer et al., 2012), while others suggest that people with dementia have a preserved ability to comprehend affective prosody and intact emotional processing (Koff et al., 1999; Kumfor and Piguet, 2012). Even less is known about expressive prosody in dementia, particularly in relation to its neural underpinnings.

This pilot study explored the expression of affective prosody and its cortical correlates in individuals with DAT using functional near-infrared spectroscopy (fNIRS). fNIRS is a noninvasive, portable neuroimaging modality well-suited for studying real-time communication in clinical populations (Kato et al., 2018; Meilán et al., 2012; Ferdinando et al., 2023). We addressed two research questions:

1. How do individuals with DAT manipulate acoustic features (e.g., pitch, rate) to express different emotions (happiness, sadness, neutral)?
2. What cortical activation patterns are associated with affective prosody production in DAT?

Given that expressive affective prosody in DAT has not been fully investigated, our hypotheses were exploratory and informed by related literature. Prior work on perception suggests that individuals with DAT may have difficulty processing affective prosody (Taler et al., 2008; Rohrer et al., 2012), and right hemisphere networks are known to play a central role in prosodic expression (Ross and Monnot, 2008). Accordingly, we expected that individuals with DAT would show minimal variation in acoustic features across emotions and no significant hemispheric differences in cortical activation during emotional speech production.

## 2 Methods

### 2.1 Participants

This study was approved by the Institutional Review Board of Northern Illinois University (HS23-0135), and all procedures and all procedures adhered to ethical standards and regulatory guidelines. Participants were eligible if they (1) were aged 65 years or older, (2) had a documented diagnosis of dementia of the Alzheimer’s type (DAT) confirmed by a board-certified neurologist or neuropsychologist, and (3) scored between 1 and 20 on the Saint Louis University Mental Status (SLUMS) examination (Morley and Tumosa, 2002), indicating mild to moderate cognitive impairment. The SLUMS is a validated cognitive screening tool that assesses orientation, memory, attention, language, and executive function, with reliability and validity comparable to or exceeding other brief cognitive assessments (Spencer et al., 2022; Dautzenberg et al., 2020). Exclusion criteria included current use of psychiatric medications or a history of comorbid neurological conditions.

Ten individuals with DAT were enrolled (4 male, 6 female; mean age = 71.5 years, SD = 6.31), all of whom self-identified as non-Hispanic White and native English speakers. Educational backgrounds varied: five had some college experience, three were high school graduates, one held an associate degree, and one a bachelor’s degree. All participants were right-handed, nonsmokers at the time of testing, and demonstrated sufficient cognitive and communicative abilities to follow multi-step instructions necessary for task compliance. Hair characteristics were inspected to ensure adequate scalp-optode contact, and fNIRS headbands were adjusted to optimize signal quality. Cognitive status was confirmed by SLUMS scores within the specified range.

This project was designed as an exploratory brief report and no demographically matched control group was included. Our focus was to establish feasibility of combining acoustic analysis and fNIRS in individuals with DAT, thereby generating preliminary data to inform larger controlled studies.

### 2.2 Procedures

After obtaining informed assent and consent from participants and their legal guardians, demographic information (e.g., age, education, race/ethnicity) was collected, and cognitive status was assessed using the SLUMS examination. Participants then completed three spontaneous speech tasks: (1) recounting a happy life event (happy), (2) describing a sad life event (sad), and (3) explaining how to make a peanut butter and jelly sandwich (neutral). For the emotional tasks, participants were prompted to recall and narrate a personally meaningful experience (e.g., “Tell me about a time when you felt very happy/sad”). This autobiographical recall method is known to reactivate emotions and their physiological correlates, which in turn influence vocal production and prosodic patterns (Murphy et al., 2021; Amir et al., 2000; Krasnodębska et al., 2024; Labov and Waletzky, 1997). For the neutral task, participants described the steps of making a peanut butter and jelly sandwich as if instructing someone unfamiliar with the procedure. Such procedural descriptions rely on overlearned, automatic routines, engage subcortical networks, and typically elicit factual, affect-free speech (Lum et al., 2012; Seger et al., 2011; Leaman and Archer, 2023). These tasks were selected to elicit naturalistic emotional and neutral speech, enhancing ecological validity compared to traditional reading or repetition paradigms (Kato et al., 2018; Meilán et al., 2012; Horley et al., 2010; Meilán et al., 2014; Testa et al., 2001; Wright et al., 2018). Speech was recorded in a quiet room using a Sony PCM-M10 digital recorder, with a 10 cm mouth-to-microphone distance at a 45-degree angle. Task order was randomized, and brief rest periods were provided between tasks. Each session lasted less than 20 minutes.

**Figure 1:**
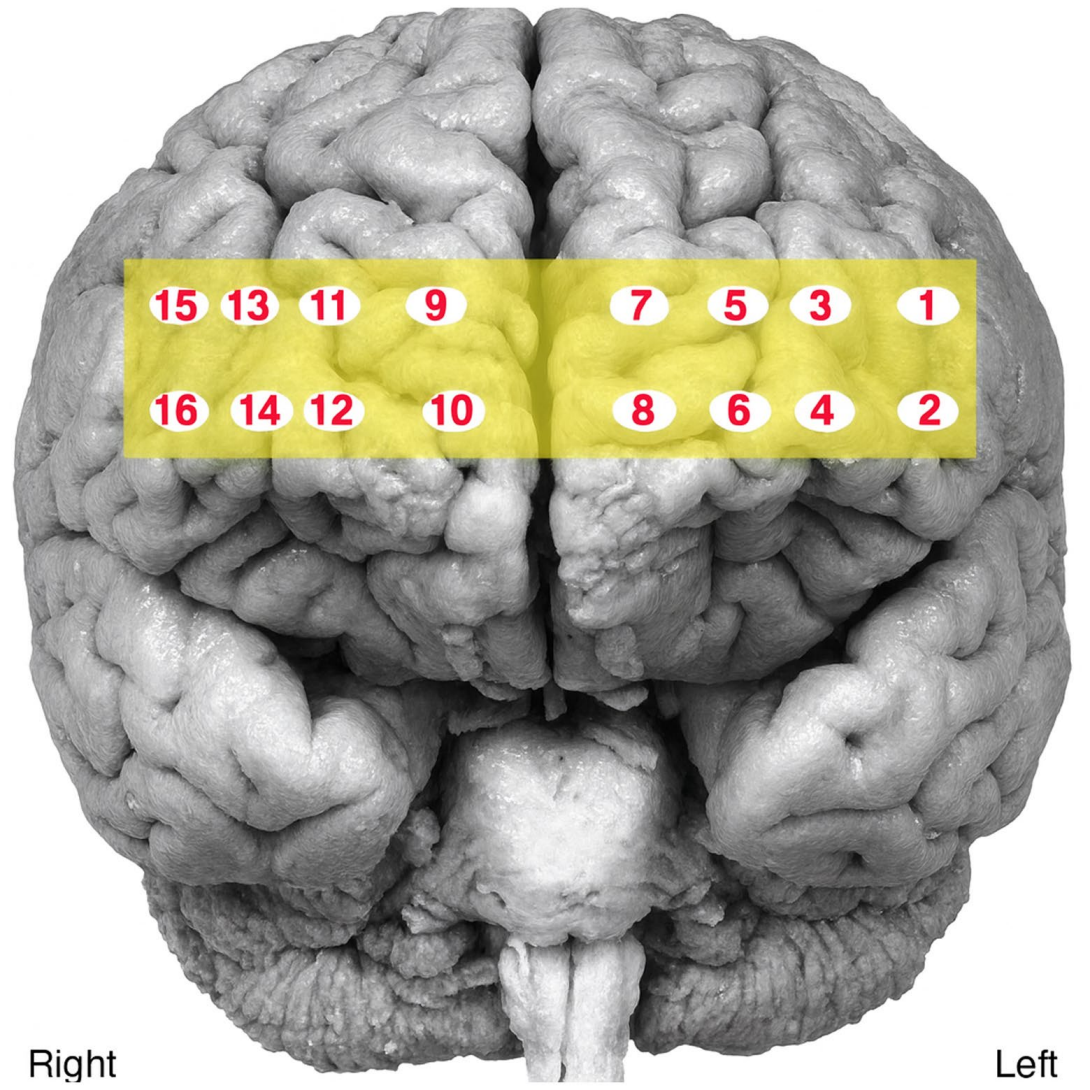
Optodes location.

Cortical activity was recorded during speech production using a 16-channel functional near-infrared spectroscopy (fNIRS) system (fNIR 400, Biopac) with two wavelengths (730 nm and 850 nm). The sensor band was placed bilaterally on the participants’ forehead to monitor prefrontal activation (Figure 1). Signals were sampled at 2 Hz and recorded using COBI Studio and fNIRSOFT software. The fNIRS signals were visually inspected to confirm proper contact of the sensor band with the participants’ foreheads and to identify any interference from hair beneath the sensors (raw signal values >4,000 mV or <400 mV). Data preprocessing included applying a 0.1 Hz low-pass filter to reduce noise. Motion artifacts and signal saturation were detected using sliding-window motion artifact rejection (SMAR), with lower and upper thresholds set at 3 and 50, respectively. A 30 s rest interval was inserted between tasks to allow hemodynamic responses to return to baseline, as recommended for fNIRS protocol (Yang and Hong, 2021).

Speech recordings were parsed into utterances by two independent raters, with disagreements resolved through discussion (initial agreement rate = 89%). Audio files were processed in Praat software to extract acoustic features of prosody (e.g., pitch, intensity, speech rate). Unvoiced segments were removed, and prosodic markers were extracted as described in Table 1. F0 was extracted in Praat using pitch-tracking thresholds set at 75–500 Hz. Speech rate was calculated as the number of syllables divided by total speech time, while articulation rate was calculated as the number of syllables divided by articulation time (excluding pauses >200 ms). Utterances were used as the unit of analysis. Speech duration was measured at the utterance level, defined as the total time from the onset of the first sound to the offset of the final sound, including all intervening pauses. All segmentation of syllables, pauses, and utterance boundaries was performed manually in Praat by 3 trained research assistants with 87% initial agreement and 100% agreement after reconciliation.

**Table 1:** Acoustic measures.

Parameter	Description	Measures	Contribution to affective prosody
Frequency	The rate at which a person’s folds vibrate (per second) that is heard as pitch	Initial, final, minimum, and maximum fundamental frequency (f0). f0 Extent.	Fear, joy, and anger are portrayed at a higher frequency than sadness (Bachorowski, 1998). f0 extent differs between happiness and fear (Paeschke and Sendlmeier, 2000). Initial f0 differs between anger and sadness (Paeschke and Sendlmeier, 2000; Sauter et al., 2010). Final f0 differs between happiness and sadness (Paeschke and Sendlmeier, 2000).
Sound pressure level (SPL)	The amplitude of speech, the acoustic measurement of loudness measured in decibel (dB)	Initial, final, minimum, and maximum SPL. SPL Extent.	Extent SPL differs between anger and happiness (Tao et al., 2006). Average SPL differs between fear and sadness (Tao et al., 2006). Initial SPL and final SPL have not been fully investigated.
Speech timing	Measures of the number of speech units of a given type produced within a given amount of time	Speech duration (the duration from initiation of first sound to termination of final sound). Speaking rate. Articulation rate.	Speech duration is longer for happiness and anger (Cole et al., 2006). Speaking rate differs among happiness, anger, sadness, fear, and neutral (Tao et al., 2006; Erdemir et al., 2018). Articulation rate is slower for negative emotions (Yildirim et al., 2004).
Pauses	Any inter-word or intra-word interval over 200 milliseconds that has no detectable speech	Pause duration (ms). Pause count per utterance.	Sad and fearful emotions are produced with more pauses, in comparison to neutral speech (Cole et al., 2006).

See Methods for details of acoustic feature extraction (e.g., F0 thresholds, rate calculations, segmentation procedures).

**Table 2:** Multinomial logistic regression predicting emotional speech from acoustic features.

Predictor	B (happy)	SE (happy)	z	*p*	B (sad)	SE (sad)	z	*p*
SumExtSyll	64.69	9915.52	0.01	0.995	63.26	9915.52	0.01	0.995
SumPause	−0.89	0.78	−1.14	0.256	−1.12	0.79	−1.42	0.155
f0i	0.018	0.0088	2.06	**0.039**	0.009	0.0088	1.03	0.302
f0f	−0.009	0.0071	−1.26	0.206	−0.003	0.0068	−0.46	0.642
dBi	0.030	0.056	0.54	0.589	0.031	0.056	0.56	0.576
dBf	−0.036	0.060	−0.60	0.546	0.012	0.062	0.19	0.853
Speech rate	−0.98	0.56	−1.75	0.080	−1.33	0.61	−2.18	**0.029**
Articulation rate	2.89	2.46	1.18	0.240	2.85	2.45	1.16	0.246
Speech	0.29	0.25	1.14	0.255	0.47	0.26	1.83	0.067
Intercept	−1.83	6.05	−0.30	0.762	−4.32	6.08	−0.71	0.477

SumExtSyll, sum of extra syllables; SumPause, sum of pauses; f0i, initial f0; f0f, final f0; dBi, initial dB; dBf, final dB; Speech, duration from initiation of first sound to termination of final sound. The bold values indicated statistically significant ones.

The fNIRS data were analyzed using the modified Beer–Lambert Law to calculate relative concentrations of oxygenated hemoglobin (HbO) and total hemoglobin (HbT) in the left and right hemispheres. Overall, channels 5, 7, 11, and 14 were determined to be of poor quality; therefore, the raw data from these channels were excluded from preprocessing and all subsequent analyses.

All statistical analyses were performed using Stata Version 17. Multinomial logistic regression was used to model emotional condition (Happy, Sad, or Neutral) as a function of continuous acoustic predictors. Paired-sample *t*-tests were conducted to assess hemispheric differences in HbO and HbT during each speech condition. To control for multiple comparisons within analytic families, the Benjamini–Hochberg false discovery rate (FDR) procedure (*α* = 0.05) was applied separately to the acoustic analyses (multinomial logistic regression predictors) and fNIRS hemisphere contrasts.

## 3 Results

The multinominal logistic regression model’s goodness-of-fit was statistically significant, χ^2^(18) = 50.92, *p* < 0.001, indicating that the model provided a better fit to the data than the null model. The log likelihood ratio test revealed a significant improvement in model fit compared to the null model, χ^2^(18) = 50.92, *p* < 0.001 (Table 1). The model accounted for approximately 26.18% of the variance in utterance type (i.e., the emotional condition of the utterances: Happy, Sad, Neutral), as indicated by the pseudo *R*^2^. For the comparison between the neutral and happy tasks, the initial fundamental frequency (f0i) was a significant predictor (*β* = 0.018, standard error [SE] = 0.009, z = 2.06, *p* = 0.039, 95% confidence intervals [CI] = [0.001, 0.035]). For the comparison between the neutral and sad tasks, the speech rate was a significant predictor (β = −1.325, SE = 0.607, z = −2.18, *p* = 0.029, 95% CI = [−2.515, −0.135]). Importantly, after FDR correction within the acoustic family, both effects (f0i for Neutral vs. Happy, and speech rate for Neutral vs. Sad) remained significant (q = 0.039 for both) (Table 2).

**Figure 2:**
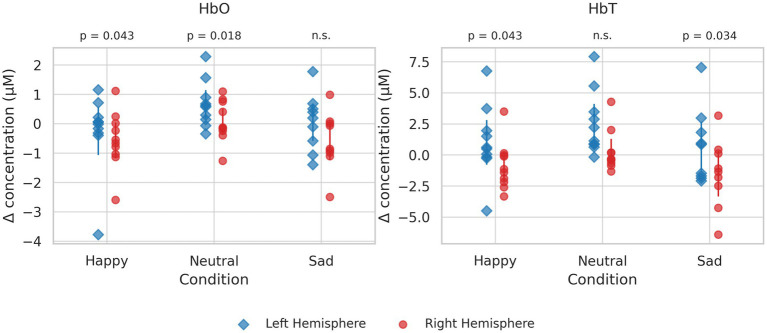
Panels show oxygenated hemoglobin (HbO, left) and deoxygenated hemoglobin (HbT, right) across three conditions (happy, neutral, sad). Each point represents an individual participant. Vertical error bars indicate group mean ± 95% confidence interval for each hemisphere within each condition. Statistical comparisons between hemispheres are displayed below the panel titles.

The HbO showed nominally significant differences between the two hemispheres during the happy (t(5) = 0.699, *p* = 0.043) and neutral tasks (t(5) = 3.459, *p* = 0.018). The HbT also significantly differed during the happy (t(5) = 2.691, *p* = 0.043) and sad (t(5) = 2.900, *p* = 0.034) speech tasks. However, none of these effects survived the FDR correction (HbO: Happy q = 0.065, Neutral q = 0.065; HbT: Happy q = 0.065, Sad q = 0.065), and thus the findings should be interpreted as preliminary. Figure 2 illustrates the fNIRS results.

## 4 Discussion

This pilot study explored the production of affective prosody and its neural correlates in individuals with DAT using fNIRS during spontaneous speech tasks. By combining acoustic and neuroimaging analyses, we aimed to assess feasibility and identify preliminary patterns of impairment relevant to affective communication in dementia.

Prosody is essential for expressing emotion and intent in everyday interactions. In neurotypical populations, specific acoustic patterns, such as higher pitch and intensity for happiness or slower speech rate for sadness, have been consistently observed across emotional states (Yildirim et al., 2004; Huttunen et al., 2019). In contrast, our findings suggest that individuals with DAT demonstrate reduced modulation of affective prosody. Only two acoustic markers (i.e., initial fundamental frequency (f0) and speech rate) emerged as significant predictors of emotional condition, with additional trends observed for speech duration. These results, which remained significant after FRD correction, align with previous work showing diminished expressive abilities in individuals with cognitive impairment (Horley et al., 2010; Haider et al., 2020; Oh et al., 2023) and underscore the challenges individuals with DAT may face in conveying emotion through speech.

With respect to neural activation, participants exhibited predominantly left-hemisphere engagement during both emotional and neutral speech tasks. However, although several contrasts reached nominal significance, none survived FDR correction, and thus these findings should be regarded as preliminary. In healthy individuals, emotional processing is typically supported by bilateral and interhemispheric networks (Frühholz and Grandjean, 2012; Grandjean et al., 2005; Meyer et al., 2004; Mitchell and Ross, 2013; Pell and Leonard, 2005; Wildgruber et al., 2004; Balconi et al., 2015), which are often disrupted in DAT (Lian et al., 2020; Qi et al., 2019). Prior neuroimaging studies of emotion processing in healthy adults have yielded inconsistent results regarding lateralization (Bendall et al., 2016; Kreplin and Fairclough, 2013; Vytal and Hamann, 2010), highlighting the complexity of these networks and the potential for further insights through dementia-focused research. Despite the preliminary nature, the findings of this study are broadly consistent with prior evidence of altered hemispheric lateralization in dementia (Amlerova et al., 2022; Cadieux and Greve, 1997; Hazelton et al., 2023; Kumfor et al., 2016) and raise the possibility that impaired interhemispheric integration may contribute to reduced affective prosody expression.

The ability to express emotion clearly is critical for meaningful communication, particularly in care settings. Impairments in affective prosody may contribute to miscommunication between individuals with dementia and caregivers, potentially affecting emotional well-being, social connection, and care outcomes. The present findings have translational implications by pointing to specific acoustic and neural features that may be targeted in future interventions to improve communicative functioning in dementia.

Several limitations must be acknowledged. First, the small sample size, homogeneity of participants, and absence of a control group substantially limit the generalizability of our findings. In particular, the fNIRS results should be considered preliminary; although several contrasts reached nominal significance, none remained significant after FDR correction, underscoring the risk of Type I error in this small sample. Without controls, dementia-related changes cannot be disentangled from those attributable to normal aging. In addition, the statistical analyses are underpowered for such a dataset, and no *a priori* power analysis was conducted given the feasibility-oriented nature of this pilot. Consistent with the exploratory nature of the study, our claims are intentionally conservative and limited to feasibility and descriptive patterns within DAT.

Second, the study was limited in the scope of emotional categories examined. Only happy and sad narratives, alongside a neutral procedural task, were included, which does not capture the full range of affective prosody relevant to everyday communication (e.g., anger, fear, surprise, or mixed emotions). Moreover, we did not perform perceptual validation of the elicited utterances (e.g., listener ratings), leaving open the question of whether the intended emotions were reliably perceived. The neuroimaging analysis was also restricted to prefrontal regions, whereas prosody production engages broader cortical and subcortical networks that were not measured here.

Third, the acoustic feature set used for this study was limited, relying mainly on pitch, intensity, timing, and pause measures. Modern approaches to acoustic analysis including spectral, cepstral, and perturbation-based features have shown sensitivity in identifying cognitive impairment (Ding et al., 2023). In addition, Measures of voice quality (e.g., jitter, shimmer, harmonics-to-noise ratio), which are important correlates of emotional expression (Schewski et al., 2025), were not evaluated here, limiting the scope of the acoustic analysis. The exclusion of these features limits the depth of interpretation and may underestimate the full extent of prosodic change in DAT.

Building on these limitations, several directions for future research are warranted. To address issues of sample size and generalizability, larger and more demographically diverse cohorts should be recruited, and matched control groups incorporated, to distinguish dementia-specific changes from those associated with normal aging. Power analyses based on effect sizes from preliminary studies should be used to guide recruitment and ensure adequately powered hypothesis-driven designs (Faul et al., 2007; Riley et al., 2020).

Expanding the scope of emotional categories beyond happiness and sadness will provide a more comprehensive view of affective prosody in dementia. Incorporating additional emotions such as anger, fear, or surprise, as well as mixed or ambiguous states, will capture the complexity of real-world communication. Perceptual validation of elicited utterances through independent listener ratings will also be critical to confirm that speech is reliably perceived as intended. At the neural level, future studies should extend cortical coverage beyond the prefrontal regions examined here, and ideally include subcortical structures, to better characterize the distributed networks supporting affective prosody.

Finally, future work should broaden the acoustic feature set beyond pitch, intensity, timing, and pauses. Incorporating spectral, cepstral, perturbation-based, and other advanced measures, and ideally integrating multimodal acoustic–linguistic analyses, may yield more sensitive and specific markers of cognitive decline. Together, these advances will strengthen the interpretability of findings and help clarify whether prosodic changes are uniquely associated with DAT or reflect broader age-related processes.

Despite these limitations, this study demonstrates the feasibility of using fNIRS to investigate affective prosody production in individuals with DAT and highlights behavioral and neural features that may underlie communication challenges in this population. These findings provide a foundation for larger-scale, hypothesis-driven studies that can support the development of targeted communication interventions and contribute to early functional markers of dementia progression.

## Data Availability

Due to the sensitive nature of the raw audio recordings and the potential risk of participant re-identification, these data cannot be made publicly available. De-identified, coded datasets underlying the analyses may be shared by the authors upon reasonable request, and decisions will be made on a case-by-case basis in accordance with institutional and ethical guidelines.
